# Expression, Purification and Characterization of a Novel Hybrid Peptide CLP with Excellent Antibacterial Activity

**DOI:** 10.3390/molecules26237142

**Published:** 2021-11-25

**Authors:** Junhao Cheng, Marhaba Ahmat, Henan Guo, Xubiao Wei, Lulu Zhang, Qiang Cheng, Jing Zhang, Junyong Wang, Dayong Si, Yueping Zhang, Rijun Zhang

**Affiliations:** Laboratory of Feed Biotechnology, State Key Laboratory of Animal Nutrition, College of Animal Science & Technology, College of Veterinary Medicine, China Agricultural University, Beijing 100193, China; chengjunhao@cau.edu.cn (J.C.); malika511@126.com (M.A.); ghn_657@cau.edu.cn (H.G.); weixubiao01@cau.edu.cn (X.W.); zhanglulu09@cau.edu.cn (L.Z.); chengqiangcool@163.com (Q.C.); zhangjing123@cau.edu.cn (J.Z.); wangjy9722@163.com (J.W.); dayong@cau.edu.cn (D.S.)

**Keywords:** hybrid peptide, antimicrobial peptide, *Escherichia coli*, fusion expression, antibacterial activity

## Abstract

CLP is a novel hybrid peptide derived from CM4, LL37 and TP5, with significantly reduced hemolytic activity and increased antibacterial activity than parental antimicrobial peptides. To avoid host toxicity and obtain high-level bio-production of CLP, we established a His-tagged SUMO fusion expression system in *Escherichia coli*. The fusion protein can be purified using a Nickel column, cleaved by TEV protease, and further purified in flow-through of the Nickel column. As a result, the recombinant CLP with a yield of 27.56 mg/L and a purity of 93.6% was obtained. The purified CLP exhibits potent antimicrobial activity against gram+ and gram- bacteria. Furthermore, the result of propidium iodide staining and scanning electron microscopy (SEM) showed that CLP can induce the membrane permeabilization and cell death of Enterotoxigenic *Escherichia coli* (ETEC) K88. The analysis of thermal stability results showed that the antibacterial activity of CLP decreases slightly below 70 °C for 30 min. However, when the temperature was above 70 °C, the antibacterial activity was significantly decreased. In addition, the antibacterial activity of CLP was stable in the pH range from 4.0 to 9.0; however, when pH was below 4.0 and over 9.0, the activity of CLP decreased significantly. In the presence of various proteases, such as pepsin, papain, trypsin and proteinase K, the antibacterial activity of CLP remained above 46.2%. In summary, this study not only provides an effective strategy for high-level production of antimicrobial peptides and evaluates the interference factors that affect the biological activity of hybrid peptide CLP, but also paves the way for further exploration of the treatment of multidrug-resistant bacterial infections.

## 1. Introduction

In the past few decades, as multidrug-resistant pathogens spread at an alarming rate, many efforts have been made to develop new therapeutic compounds that can be used to safely treat infectious diseases [1,2,3,4]. Antimicrobial peptides (AMPs) are a potential alternative to antibiotic treatment [5,6]. They are amphipathic, cationic, and small polypeptides [7]. AMPs are widely distributed in nature and play an important role in resisting pathogen infection [8,9,10]. AMPs usually destroy the lipid bilayer of microorganisms through physical action and induce the outflow of cell contents to produce antibacterial effects [11]. AMPs exhibit antimicrobial activity through various mechanism models, common models are “barrel-stave”, “carpet” and “toroidal-pore” [12,13,14]. These sterilization mechanisms can reduce the production of resistant bacteria. Therefore, AMPs show great promise as a new type of antibacterial agent, which can kill antibiotic-resistant bacterial pathogens. 

CM4, isolated from *Bombys mori* (a Chinese domestic silk moth), is an α-helical cationic antibacterial peptide, exhibiting a broad range of antimicrobial activity [15]. It kills pathogenic bacteria by destroying the integrity of cell membranes [16,17]. LL37, a kind of antibacterial peptide of the cathelicidin family in humans, plays an important role in the process of antibacterial infection and immune regulation [18,19]. However, LL37 also exhibits unwanted hemolytic activity to host cells, which greatly limits its clinical application [20]. TP5 is a pentapeptide derived from the thymus hormone, which regulates and enhances the immune function of human cells [21,22,23]. Hybridization of parent peptides is an effective strategy to generate new AMPs, if properly designed, this strategy can minimize cytotoxicity and significantly improve antibacterial activity [24,25,26].

Despite the increasing demand for clinical applications of AMPs, the production of AMPs relies on high-cost chemical synthesis. Therefore, some recombinant expression strategies have been applied to produce antimicrobial peptides [27,28,29]. *Escherichia coli* (*E. coli*) has been widely used for recombinant expression of various foreign genes. The advantages of this system include high yield, relatively simple genetic manipulation, and relatively low cost [30,31]. However, the toxicity and insolubility (precipitation in inclusion-body) of AMPs to host bacteria limits their recombinant expression. In addition, degradation by proteases is also a challenge for the recombinant expression of antimicrobial peptides. Fusion expression strategy may be the solution to this problem because chaperone proteins can hide the toxicity of antimicrobial peptides to the host and can reduce protease degradation. There are a number of fusion choices, including GST, MBP, Trx, SUMO, etc. [32,33,34,35]. Among these, SUMO is the most effective chaperone protein in the *E. coli* expression system because it can increase the solubility of the protein, mask the possible toxicity of antimicrobial peptides to the host and reduce the degradation of the target protein by proteases [36].

In the current study, we report on a novel hybrid peptide CLP with the combination of CM4 (1–8), LL37 (17–30) and TP5 (1–5) for its improving antibacterial activity and minimizing cytotoxic effects. Importantly, CLP can be expressed as a fusion protein, cleaved, and purified from *E. coli*, with a yield of 27.56 mg/L, and its antimicrobial activities, stability, and hemolytic activity were further examined.

## 2. Results

### 2.1. Construction of Recombinant Expression Plasmids

In our previous work, we constructed an effective plasmid construction strategy, using Golden Gate cloning technology to clone the target gene into the pET-Amp plasmid [37]. The diagrammatic sketch of the construction of the recombinant expression plasmid was shown in Figure S1. The expression plasmid pET-SUMO-TEV-CLP was constructed by synthesizing the His-SUMO-TEV-CLP gene and cloning it into pET-Amp. His-tag was connected to the N terminal of SUMO. TEV protease cleavage site between SUMO and CLP. The correct plasmid was confirmed by Sanger sequencing.

### 2.2. Expression of SUMO-TEV-CLP

**Figure 1:**
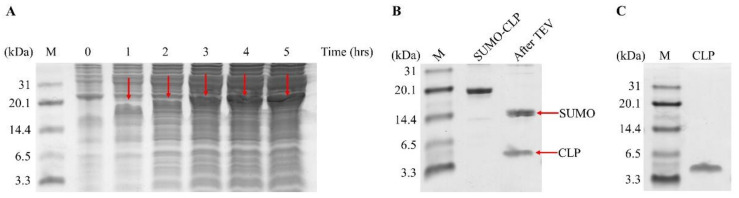
The expression and purification of CLP. (**A**) Tris-Tricine-SDS-PAGE detection of cell lysate supernatant after IPTG induction for 1 to 5 h. (**B**) Tris-Tricine-SDS-PAGE detection of purified SUMO-TEV-CLP and its product cleaved by TEV protease. (**C**) Tris-Tricine-SDS-PAGE detection of purified CLP.

The pET-SUMO-TEV-CLP plasmid was transformed into *Escherichia coli* BL21 (DE3) and induced by 0.5 mM IPTG in LB medium. After cell lysis and centrifuge, the recombinant peptide could be detected by 15.5% Tris-Tricine-SDS-PAGE. The result was shown in Figure 1A, a band with a molecular weight of about 18 kDa was observed. After IPTG induction, the maximum amount of SUMO-TEV-CLP was observed at 4 and 5 h.

### 2.3. Purification and Cleavage of SUMO-TEV-CLP

The His-tagged SUMO-TEV-CLP was purified by immobilized metal ion affinity chromatography (IMAC). Tris-Tricine-SDS-PAGE SDS analysis showed that a clean band was observed at 18 kDa (Figure 1B, lane 2). The recombinant CLP was cleaved from SUMO-TEV-CLP by TEV protease at 16 °C for 6 h. The results were analyzed by Tris-Tricine-SDS-PAGE and most SUMO-TEV-CLP has been cleaved in this condition (Figure 1B, lane 3).

### 2.4. Purification of Recombinant CLP

**Figure 2:**
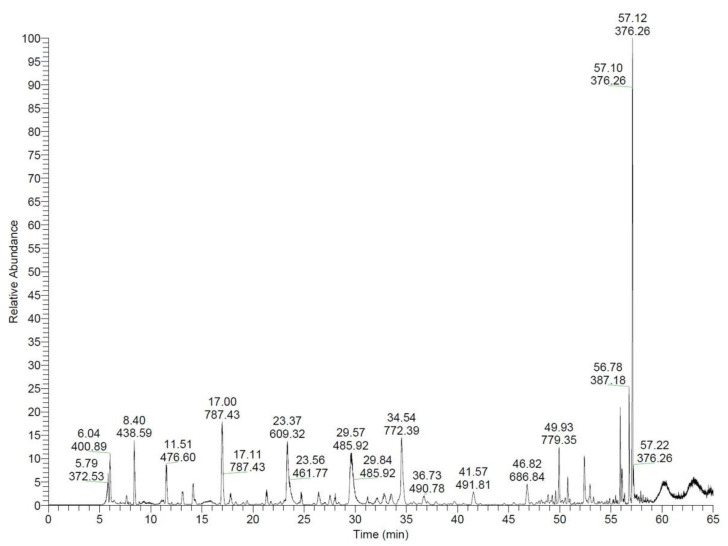
Analysis of purified CLP using electrospray ionization–mass spectrometry.

**Figure 3:**
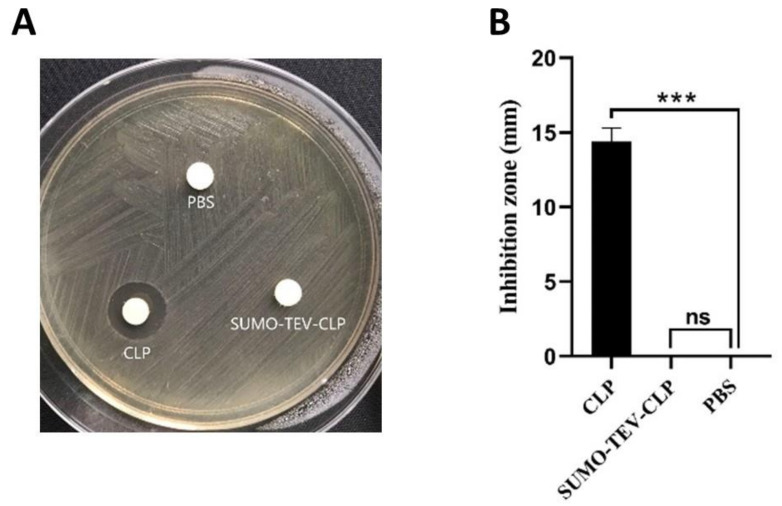
The antimicrobial activity of recombinant CLP against ETEC K88 at 37 °C for 12 h. (**A**) Inhibition zone of CLP and SUMO-TEV-CLP on ETEC K88. (**B**) Statistical analysis of the inhibition zone of ETEC K88 by CLP and SUMO-TEV-CLP. CLP represents recombinant CLP; SUMO-TEV-CLP represents the recombinant fusion protein SUMO-TEV-CLP; PBS represents sodium phosphate buffer was the negative control. One-way analysis of variance (ANOVA) and Dunnett’s multiple comparisons test were used for statistical analysis. *** *p* < 0.01 indicates a significant difference compared with the PBS control. Finally, ns indicates no significant difference.

The recombinant CLP present in the digestion solution was purified again using IMAC. Both TEV and SUMO contain His-tags, so cleaved CLP was presented in the flow-through. The results were analyzed by Tris-Tricine-SDS-PAGE to verify the successful isolation of recombinant CLP in the flow-through fraction (Figure 1C). The final yield of recombinant CLP was 27.56 mg/L. The recombinant CLP was identified by electrospray ionization–mass spectrometry (ESI-MS). The results were shown in Figure 2, the exact and predicted molecular weights of CLP are 4628.56 Da and 4628.62 Da, respectively. In order to study the antimicrobial activities of purified CLP, the inhibition zones of recombinant CLP against Enterotoxigenic *Escherichia coli* (ETEC) K88 were measured by Kirby–Bauer test method (Figure 3). The results clearly showed that the recombinant CLP significantly inhibited the growth of ETEC K88 compared to the control group and the recombinant SUMO-TEV-CLP.

### 2.5. Antimicrobial Activities of CM4, LL37, TP5, Recombinant CLP(rCLP) and Synthesized CLP (sCLP)

The minimum inhibitory concentrations (MICs) of CM4, LL37, TP5, rCLP and sCLP were examined through microbroth dilution method (Table 1). The results showed that the rCLP greatly improved the antibacterial activity of the parent peptide. The MICs of rCLP (2–8 µg/mL) much lower than CM4 (8–256 µg/mL), LL37 (16–32 µg/mL) and TP5 (over 512 µg/mL) for all the three reference strains. Additionally, the recombinant and synthesized CLP exhibited the same antibacterial activity against all three reference strains.

### 2.6. Recombinant CLP Induces Bacterial Membrane Permeabilization

**Figure 4:**
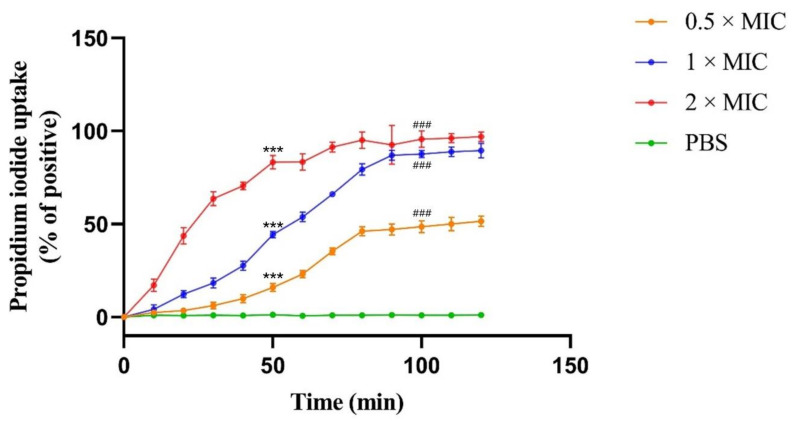
The uptake of propidium iodide (PI) by ETEC K88 under the treatment of purified CLP. The reference fluorescence (100%) was samples treated with 0.05% SDS, and PBS treatment was as a negative control. 0.5 × MIC, 1 × MIC and 2 × MIC of purified CLP against ETEC K88 were at concentrations of 1, 2 and 4 µg/mL. Data shown are averages over 3 independent replicates. The standard deviation was shown by the error bars. One-way analysis of variance (ANOVA) and Dunnett’s multiple comparisons test was used for statistical analysis. *** *p* < 0.01 and ^###^ *p* < 0.01 indicate a significant difference compared with the PBS control at 50 minutes and 100 minutes, respectively.

The propidium iodide (PI) cannot pass through the intact bacterial envelope, therefore, the uptake of PI indicates the degree of bacteria membrane damage. A PI uptake assay was performed to explore the effect of recombinant CLP on bacterial membranes. The results showed that recombinant CLP induced a significant increase in PI fluorescence in ETEC K88 (Figure 4). There was a clear correlation between recombinant CLP concentrations and PI fluorescence. Specifically, treated with recombinant CLP at concentrations of 0.5, 1 and 2 × MIC for 50 min, the uptake of PI fluorescence by ETEC K88 increased by 16.0%, 44.3% and 83.3%, respectively. Furthermore, after 120 min of treatment, the fluorescence increased by 51.5%, 89.5% and 97.0%, respectively. Compared with the PBS control, the fluorescence intensities of 0.5, 1 and 2 × MIC were significantly different at 50 min and 100 min, respectively.

**Figure 5:**
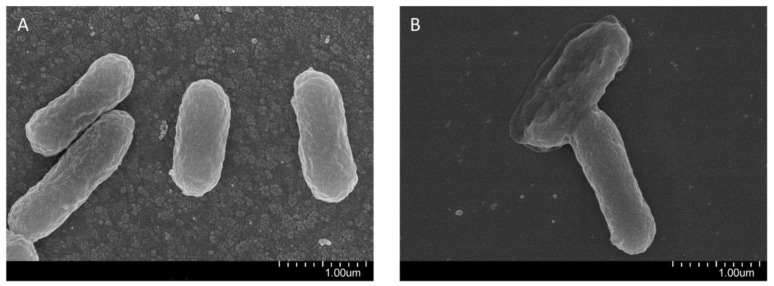
Scanning electron microscope micrographs of ETEC K88 treated by recombinant CLP. (**A**) No peptide (Control). (**B**) Treated with 1 × MIC recombinant CLP for 2 h. Scale bar, 1.0 µm.

In addition, a scanning electron microscope was applied to observe the morphology of ETEC K88 when exposed to 2 µg/mL recombinant CLP for 2 h (Figure 5). The results showed clear differences in the membrane morphology of untreated and CLP-treated ETEC K88. The untreated bacterial membrane is smooth and intact, however, treatment with 1 × MIC of recombinant CLP for 2 h damaged the integrity of the bacterial cell membrane, which may create pores on the bacterial cells.

### 2.7. Effect of pH, Temperature, and Proteinases on Recombinant CLP Activity

**Figure 6:**
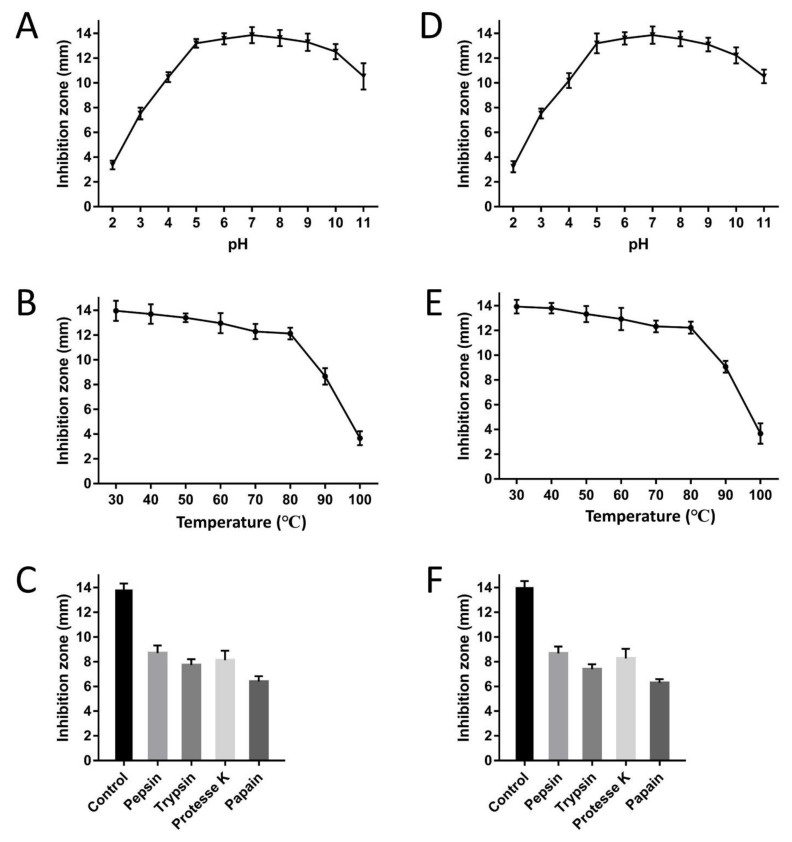
The influence of pH, temperature and protease treatment on the antibacterial activity of recombinant CLP (**A**–**C**) and synthesized CLP (**D**–**F**). (**C**) The effects of different protease treatments on the antibacterial activity of recombinant CLP. PBS was employed as a control. ETEC K88 was used as the reference strain. Data shown are averages over 3 independent replicates. The standard deviation was shown by the error bars.

Inhibition zone assays were used to investigate the effects of pH, temperature and proteolytic enzymes on the action of CLP against ETEC K88. To investigate thermal stability, the activity of recombinant CLP was examined after treatment at different temperatures. The results confirmed that recombinant CLP was thermally stable and its antimicrobial activity was almost completely retained even when exposed to 70 °C for 30 min. However, temperatures above 70 °C significantly reduced the activity of recombinant CLP (Figure 6A). In addition to thermal stability, the influence of pH on the antimicrobial activity of the CLP was also evaluated in various pH values (2.0 to 11.0). The results showed that recombinant CLP could maintain their antimicrobial activity at different pH values. More interestingly, the recombinant CLP maintained more than 74.8% of its activity throughout a pH range of 4.0 to 9.0 (Figure 6B). In the present study, the results showed in Figure 6C, under the action of various proteases (pepsin, papain, trypsin and proteinase K), recombinant CLP maintained above 46.2% of functional activity. In addition, compared with recombinant CLP, synthesized CLP maintained almost the same stability under the same temperature, pH value, and enzyme treatment as described above.

### 2.8. Hemolytic Activity of CLP

**Figure 7:**
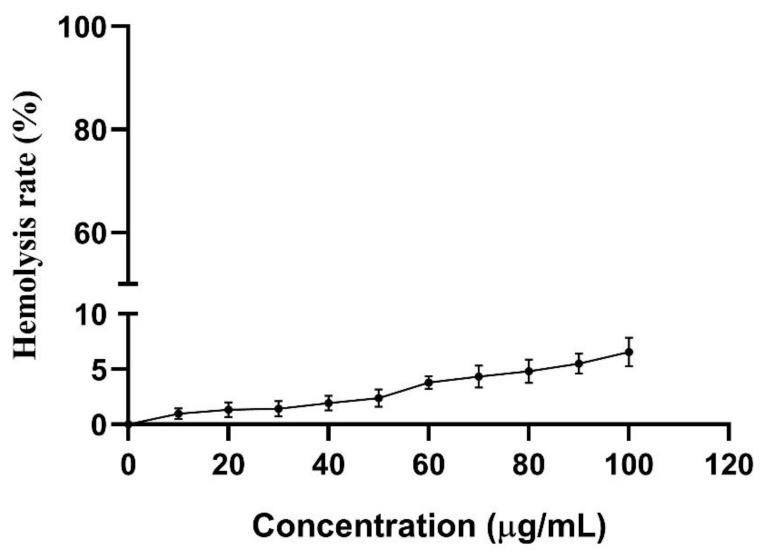
Hemolytic effects of purified hybrid peptide CLP against sheep RBCs. Data shown are averages over 3 independent replicates. The standard deviation was shown by the error bars.

The low hemolytic activity of antimicrobial peptides on mammalian red blood cells is very important in clinical applications. The hemolytic activity of the recombinant CLP against sheep erythrocyte cells was evaluated using serial peptide concentrations (from 10 to 100 µg/mL), and the results were shown in Figure 7. The recombinant CLP exhibited very low levels of hemolytic activity against sheep erythrocyte cells. When the concentration of CLP was 100 µg/mL, the hemolysis of CLP against sheep blood cells was less than 6.6%. This is much lower than previously reported that at the concentration of 90 µg/mL of LL37, the hemolysis of LL37 against human erythrocytes is about 30% [38].

## 3. Discussion

The emergence of more and more multidrug resistant strains has threatened human health worldwide. We will face a life-threatening fate unless new antimicrobial agents are promptly developed. AMPs are effective antibacterial agents as they play essential roles against harmful pathogens of vertebrates [6]. However, several AMPs, like LL37 and melittin, exhibit unwanted hemolytic activity against red blood cells [20,39], which greatly limits their application in internal medicine. Hybridization of the natural peptides has been a successful technique to construct novel peptides with improved antimicrobial activity and minimized cytotoxicity [1,2]. In addition, effective production approaches of AMPs are necessary for clinical and industrial applications. Many manufacturing methods have been used to produce AMPs, such as extraction from nature and liquid-phase peptide synthesis. However, natural extraction and liquid-phase peptide synthesis are inefficient and costly. The recombinant expression method provides a solution for the large-scale production of AMPs. The fusion expression of AMP and chaperone protein has been proved to be a successful strategy in the *E. coli* expression system, which can efficiently eliminate the possible toxicity of antimicrobial peptides to the host, increase the solubility of the AMPs, and reduce the degradation of the AMPs by proteases [40,41,42].

In the current study, SUMO-TEV-CLP was recombinantly expressed in *E. coli*. Recombinant antimicrobial peptide maintains its natural N-terminus, which is the key to its antibacterial activity [43]. Therefore, the recombinant CLP must be released from the fusion protein SUMO-TEV-CLP. In this study, SUMO-TEV-CLP was cleaved by TEV protease. After purification with the HisTrap HP column, a yield of 27.56 mg/L recombinant CLP was recovered in the flow-through, which was higher than 17.34 mg/L C-L [1], 7.3 mg/L A20L [44] and 0.3 mg/L LL37 [45]. Our Tris-Tricine-SDS-PAGE findings revealed that the recombinant CLP had a size of around 4.7 kDa, which was closed to the projected molecular weight.

In this study, our antimicrobial activity results showed that the purified CLP has strong antibacterial activity against both gram+ and gram- bacteria. More encouragingly, the recombinant CLP showed higher antimicrobial activity than parental peptides (CM4, LL37 and TP5) and similar antimicrobial activity to synthesized CLP. CLP is a cationic antimicrobial peptide with a theoretical isoelectric point of 11.11. We speculate that the antibacterial activity of CLP depends on electrostatic interactions between the negatively charged domain of phospholipids in the bacterial cell membrane and the cationic domain of CLP. In addition, the antibacterial activity of CLP may be related to the electrostatic interaction between lipopolysaccharides in the bacterial cell wall and CLP. The antibacterial activity of CLP against more strains (such as colistin-resistant *Escherichia coli*) and the cell selectivity of CLP will be studied in our future work. The antibacterial activity of CLP was further explored through the PI uptake assay. Our findings implicate that CLP-induced ETEC K88 membrane permeated in a concentration-dependent manner. The PI uptake enhances with increased concentrations of CLP at the same incubation time. Scanning electron microscopy (SEM) was applied to further confirm the antimicrobial activity of CLP. The findings revealed that CLP damages the cell membrane of ETEC K88, leading to changes in the permeability of the cell membrane. These findings indicated that CLP is a propitious peptide that can kill bacteria by destroying the integrity of the cell membrane. In addition, we need to further clarify the antibacterial properties of CLP through immunofluorescence images of bacterial cells cultured on different substrates in our future work.

It is critical to evaluate the interference factors that affect the biological activity of hybrid peptide CLP when applied to humans or livestock. In this study, the tolerance of CLP to pH, temperature and protease was tested. The results showed that it has higher temperature tolerance at different temperatures (from 30 to 70 °C). Even more encouraging, the hybrid peptide CLP maintained most of its bioactivity at 70 °C. The pH tolerance test results revealed substantial activity throughout a pH range of 4.0 to 9.0. High pH tolerance allows CLP to exert antibacterial activity in the large intestine [46]. However, the antibacterial activity of CLP may drop sharply in the stomach because its pH is lower than 4.0 [46]. The antibacterial activity is affected by the electrostatic interaction between the cationic domain of the cationic amphiphilic peptide and the negatively charged group of the bacterial plasma membrane. We speculate that pH may affect the antibacterial activity by affecting the charge state of CLP amino acids, which requires further research in our future work. 

It is necessary to evaluate the effects of proteases on the antimicrobial activity of CLP, such as pepsin, papain, trypsin and proteinase K [47]. The results of this study indicated that CLP can partially tolerate proteolytic digestion. However, when CLP was treated with papain, the antibacterial activity was reduced by nearly half, which was consistent with previous reports that protease reduced the antibacterial activity of C-L [1]. 

Finally, the hemolysis of the CLP on sheep red blood cells was tested. Compared with PBS control, CLP has very low hemolytic activity. In the next work, we will further clarify the immunogenicity and toxicity of CLP to healthy mammalian cells through in vivo and in vitro experiments. In general, these studies indicated that the novel hybrid peptide CLP is a promising peptide with prospective applications in the feed and medical industries.

## 4. Materials and Methods

### 4.1. Strains and Plasmid

The *Escherichia coli* BL21 (DE3) and *Escherichia coli* DH5α were purchased from Tiangen (Beijing, China). The pET-Amp plasmid was saved in our laboratory. Reference strains *Staphylococcus aureus* CVCC 1882 and *Escherichia coli* K88 were stored in our laboratory. *Pseudomonas aeruginosa* 337005 was purchased from BeNa Culture Collection (Beijing, China).

### 4.2. Regents and Enzymes

BsaI-HFv2 restriction enzyme, T4 DNA ligase and Q5 High-Fidelity DNA Polymerases were obtained from NEB (New England Biotech, Hitchin, UK). TEV protease and ultra-low molecular weight protein marker were purchased from Solarbio (Beijing, China).

### 4.3. Synthesis of CLP

Using APD2, created a hybrid peptide CLP from the parent peptides (CM4, LL-37, and TP5), taking into account structure-activity linkages. Then, CLP was synthesized by 9-fluorenylmethoxycarbony solid-phase synthesis chemistry and purified by a reverse-phase semi-preparative HLPC.

### 4.4. Construction of Expression Vectors

The gene of His-SUMO-TEV-CLP was synthesized by Genewiz (Suzhou, China) and cloned into pUC57. Primer F1 (5′- AAAGGTCTCAATCCAGCCATCATCATCATCATCACAGCAGC-3′) and Primer R1 (5′- AAAGGTCTCAAGCCTCAGGTAACATCCTTACGTTCAGTACGAG-3′) were used to amplify the gene of His-SUMO-TEV-CLP. The PCR was performed in a thermocycler, 98 °C for 30 s, then 35 cycles (98 °C for 10 s, 55 °C for 30 s and 72 °C for 30 s), then 72 °C for 2 min. 2% gel electrophoresis was used to verify amplified fragments. DNA gel extraction kit (Omega, Norwalk, CT, USA) was used to purify amplified fragments. The amplified DNA fragments and pET-Amp plasmid were then digested/ligated using Golden Gate cloning reaction to form the pET-SUMO-TEV-CLP expression plasmid. The reaction mixture consisted of 20 ng PCR products and pET-Amp plasmid, 1.6µL BsaⅠ-HFv2 restriction enzyme, 0.4 µL T4 DNA ligase buffer in a 20 µL total reaction volume. The reaction products were performed in a thermocycler, 37 °C for 30 min, followed by 16 cycles of 37 °C for 10 min and 16 °C for 5 min, followed by 16 °C for 60 min and 80 °C for 6 min. The Golden Gate reaction mixtures were integrated into *E. coli* DH5α. Then, strains were plated on Luria-Bertani agar with 50 µg/mL kanamycin and incubated at 37 °C overnight, the clones grown on the plate were plated on the Luria-Bertani agar with 50 µg/mL ampicillin. Positive clones were grown in Luria-Bertani agar with 50 µg/mL kanamycin, but not in Luria-Bertani agar with 50 µg/mL ampicillin. TIANprep Midi Plasmid Kit (Tiangen, Beijing, China) was used to isolate plasmid DNA and then the plasmid was confirmed by Sanger sequencing.

### 4.5. Expression of SUMO-TEV-CLP

The expression plasmids were transformed into *E. coli* BL21 according to the manufacturer’s instructions. The recombinant expression strains were cultivated in Luria-Bertani broth at 37 °C with 50 µg/mL kanamycin. Then, 0.5 mM IPTG was used to induce the expression of SUMO-TEV-CLP for 5 h. Bacterial cells were collected by centrifuging at 10,000× *g* for 10 min. Buffer A (pH 8.0, 500 mM NaCl, 20 mM Tris/HCl, 5 mM imidazole) was used for re-suspended bacterial pellets. Subsequently lysed by a low-temperature ultrahigh-pressure continuous-flow cell crusher (JN-02C, JNBIO, China) at 100 MPa. Tris-Tricine-SDS-PAGE analysis was then used to analyze the centrifuged supernatant after cell lysis.

### 4.6. Purification of SUMO-TEV-CLP

The fusion peptide was purified with an ÄKTA fast-performance liquid chromatography (FPLC) system (GE Healthcare Bio-Sciences, Uppsala, Sweden). The clarified lysate flowed through the HisTrap HP column (GE Healthcare, Chicago, IL, USA). Then, eluted with a gradient of 10% to 100% buffer B (pH 8.0, 500 mM NaCl, 20 mM Tris/HCl, 500 mM imidazole). Tris-Tricine-SDS-PAGE was used to evaluate the eluted fractions, which were subsequently concentrated using Amicon Ultra centrifugal filters (Millipore, Burlington, MA, USA). The fusion protein was dialyzed at 4 °C overnight with 6 L PBS.

### 4.7. Cleavage and Purification of the Recombinant CLP

SUMO-TEV-CLP was cleaved by TEV protease (Solarbio, Beijing, China) at 16 °C for 6 h. The SUMO part with His-tag and the small CLP were then separated using a 5 mL HisTrap HP column. Tris-Tricine-SDS-PAGE was used to detect the collected small CLP in the flow-through. The reversed-phase high performance liquid chromatography (RP-HPLC) was used to further detect the purity of collected CLP. The bicinchoninic acid (BCA) method was used to quantify purified CLP, and the experiment was performed in three independent replicates.

### 4.8. Antibacterial Activity

#### 4.8.1. Inhibition Zone

The Kirby–Bauer diffusion method was used to detect the antibacterial activity of purified CLP. ETEC K88 was used as a reference strain. The bacteria were cultivated for 12 h at 37 °C in Mueller-Hinton Broth (MH, Solarbio, Beijing, China). Then, the bacterial solution was diluted to a concentration of 5 × 10^6^ CFU/mL. Then, 150 µL diluted bacterial solution was coated on MH solid medium. The filter paper disks containing 50 µL of purified SUMO-TEV-CLP and CLP were placed on the MH agar and cultivated at 37 °C for 12 h. Finally, the vernier caliper was used to measure the diameter of the inhibition zone. Then, 50 µL PBS was used as a negative control. The experiment was performed in three independent replicates.

#### 4.8.2. Minimal Inhibitory Concentrations

A micro-dilution method was used to measure the minimal inhibitory concentrations (MICs) of CM4, LL37, TP5 and CLP. The reference strains *P. aeruginosa* 337005, *S. aureus* 1882 and ETEC K88 were cultivated at 37 °C for 12 h. Then, the bacterial solution was diluted to a concentration of 5 × 10^6^ CFU/mL. Then, 180 µL MH broth per well were added to the microtiter plate (Corning, Corning, NY, USA), and then 2 µL of the diluted bacterial solution was dispensed into each well. Peptides were then serially diluted with Milli-Q water, and 20 µL dilutions were added to the wells. Then, 2 µL Milli-Q water instead of 2 µL diluted bacterial solution was used as a blank control, and 20 µL Milli-Q water instead of 20 µL peptide dilution was used as a growth control. After culturing for 12 h at 37 °C, the MIC of the reference strain was observed, and the experiment was performed in three independent replicates.

#### 4.8.3. Propidium Iodide Uptake

PI uptake method was performed to determine whether CLP could affect the cytoplasmic membrane of ETEC K88. PI can stain DNA, but cannot penetrate the complete cell membrane. Therefore, the fluorescence intensity of PI is positively correlated with the degree of cell membrane damage. The reference strain ETEC K88 was cultured at 37 °C for 12 h, and then diluted to 5 × 10^5^ CFU/mL. Then, 10 µg/mL PI (Thermo Fisher, Waltham, MH, USA) was added to the diluted bacterial solution. The bacterial solution was dispensed into a microtiter plate (Corning, Corning, NY, USA), Then, CLP with different concentration gradients was added to the microtiter plate. The fluorescence intensity was measured using a microplate reader (Synergy H1, Bio Tek, Winooski, VT, USA) at the excitation wavelength and emission wavelength of 535 nm and 617 nm, respectively. During the continued 120 min reaction, the fluorescence values were monitored every 10 min. The PBS was a negative control, and 0.05% SDS was a positive control. 

#### 4.8.4. Scanning Electron Microscopy 

Scanning electron microscopy (SEM) was used to observe the damage of CLP to bacterial cell membranes. ETEC K88 was incubated at 37 °C for 12 h and then diluted with PBS to 5 × 10^5^ CFU/mL. The diluted ETEC K88 was treated with 1 × MIC CLP at 37 °C for 2 h. The CLP-treated ETEC K88 was washed with PBS and treated with 2.5% glutaraldehyde overnight. After dehydrating with alcohol and drying for gold coating, the samples were imaged by Quanta 200 (FEI).

### 4.9. Stability Assessment of CLP 

The stability of rCLP and sCLP in a wide pH range (2.0 to 11.0 for 2 h) and different temperatures (30 to 100 °C for 30 min) was assessed. The sensitivity of CLP to pepsin, papain, trypsin and proteinase K was also evaluated by adding 10 µg/mL of different proteases. The Kirby–Bauer diffusion method was used to detect the stability of rCLP and sCLP. The reference strain was ETEC K88.

### 4.10. Hemolytic Assay

A hemolysis test was used to measure the hemolytic activity of CLP on sheep red blood cells (SRBC). Fresh sheep RBCs (Solarbio, Beijing, China) were washed twice with PBS. The 180 µL sheep RBCs were added to the microtiter plate. Milli-Q water was used to dilute CLP to a series of concentration gradients. Then, 20 µL each concentration of CLP was dispensed into the microtiter plate and cultured at 37 °C for 1 h. The supernatants were aspirated after centrifuged at 1000× *g* for 5 min. Hemolytic activity was evaluated at 414 nm absorbance (Abs). Then, 0.1% Triton X-100 and PBS were used as the positive control and negative control. The formula for calculating the % of hemolysis was as follows:

$$\%\;\mathrm{hemolysis}=\frac{\left(\mathrm{Abs}414\;\mathrm{of}\;\mathrm{sample}-\mathrm{Abs}414\;\mathrm{of}\;\mathrm{negative}\;\mathrm{control}\right)\times 100}{\mathrm{Abs}414\;\mathrm{of}\;\mathrm{positive}\;\mathrm{control}-\mathrm{Abs}414\;\mathrm{of}\;\mathrm{negative}\;\mathrm{control}}$$

## 5. Conclusions

In conclusion, an efficient method for expressing and producing a new hybrid peptide CLP in *E. coli* has been devised. The SUMO fusion expression method was successfully applied to mask the toxicity and reduce the degradation of recombinant CLP. After successful digestion by TEV protease and purification by nickel column, a high yield of recombinant CLP with potent antimicrobial activity was achieved. The purified recombinant CLP showed strong bactericidal activity against ETEC K88 by impairing its cell membrane integrity. More encouragingly, recombinant CLP exhibited the same antibacterial activity as synthesized CLP and was significantly better than the parental peptides. The recombinant CLP showed antibacterial activity at different temperatures with a wide range of pH conditions. In addition, the recombinant CLP was, to some extent, resistant to proteolytic digestion by the tested proteases. Additionally, CLP has almost no hemolytic effect on SRBC. Our research results showed that the SUMO fusion technology in *E. coli* has great advantages in the high-level expression of antimicrobial peptides that are toxic to the host. In addition, as a new hybrid peptide with superior antibacterial characteristics, CLP has considerable development potential as an antimicrobial agent against multidrug-resistant pathogens in the future.

## Figures and Tables

**Table 1:** Minimum inhibitory concentrations (MICs) of CM4, LL37, TP5, recombinant CLP and synthesized CLP against reference bacterial strains.

Indicated Strains	MIC (µg/mL)				
	LL37	CM4	TP5	rCLP	sCLP
*P.aeruginosa* 337005	32	32	>512	2	2
*S. aureus* 1882	32	256	>512	8	8
ETEC K88	16	8	>512	2	2

MICs, minimal inhibitory concentrations; rCLP, recombinant CLP; sCLP, synthesized CLP; *P. aeruginosa*, *Pseudomonas aeruginosa*; *S. aureus*, *Staphylococcus aureus*; ETEC K88, Enterotoxigenic *Escherichia coli* K88.

## Data Availability

Data can be requested from the authors.

## Supplementary Materials

The following are available online, Figure S1: The diagrammatic sketch of the construction of the recombinant expression plasmid.

## Sample Availability

Samples of the compounds can be requested from the authors.
