# Supplementary material for: Expression, Purification and Characterization of a Novel Hybrid Peptide CLP with Excellent Antibacterial Activity
Source: Molecules. 2021 Nov 25;26(23):7142. doi: 10.3390/molecules26237142 (PMC8659006; doi:10.3390/molecules26237142)
Supplement: Supplementary file 1 [file molecules-26-07142-s001.zip › molecules-1454534-supplementary.pdf]

molecules

Supplementary File

Expression, Purification and Characterization of a Novel Hybrid Peptide CLP with Excellent Antibacterial Activity

Junhao Cheng, Marhaba Ahmat, Henan Guo, Xubiao Wei, Lulu Zhang, Qiang Cheng, Jing Zhang, Junyong Wang, Dayong Si, Yueping Zhang * and Rijun Zhang *

Laboratory of Feed Biotechnology, State Key Laboratory of Animal Nutrition, College of Animal Science & Technology, College of Veterinary Medicine, China Agricultural University, Beijing 100193, China; chengjunhao@cau.edu.cn (J.C.); malika511@126.com (M.A.); ghn_657@cau.edu.cn (H.G.); weixubiao01@cau.edu.cn (X.W.); zhanglulu09@cau.edu.cn (L.Z.); chengqiangcool@163.com (Q.C.); zhangjing123@cau.edu.cn (J.Z.); wangjy9722@163.com (J.W.); dayong@cau.edu.cn (D.S.)

* Correspondence: zhangyueping@cau.edu.cn (Y.Z.); zhangrj621@126.com (R.Z.); Tel.: +86-10-6273-1208

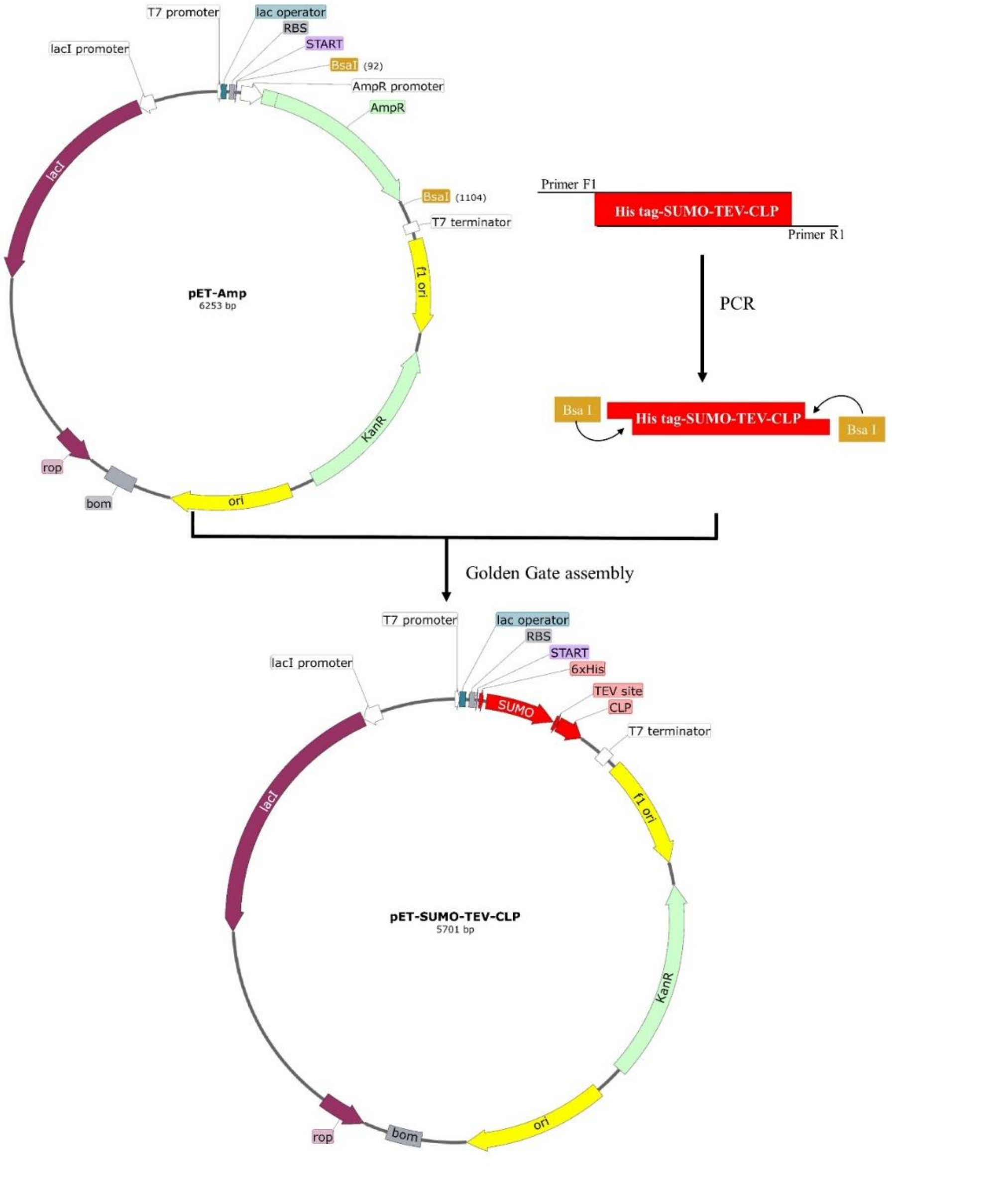

Figure S1. The diagrammatic sketch of the construction of the recombinant expression plasmid.
